# A Role of hIPI3 in DNA Replication Licensing in Human Cells

**DOI:** 10.1371/journal.pone.0151803

**Published:** 2016-04-08

**Authors:** Yining Huang, Aftab Amin, Yan Qin, Ziyi Wang, Huadong Jiang, Lu Liang, Linjing Shi, Chun Liang

**Affiliations:** 1 Biomedical Research Institute, Shenzhen-PKU-HKUST Medical Center, Shenzhen, China; 2 Division of Life Science, Center for Cancer Research and State Key Lab for Molecular Neuroscience, Hong Kong University of Science and Technology, Hong Kong, China; 3 HKUST Fok Ying Tung Research Institute, Guangzhou, China; 4 Intelgen Ltd., Hong Kong-Guangzhou-Foshan, China; University of Minnesota, UNITED STATES

## Abstract

The yeast Ipi3p is required for DNA replication and cell viability in *Sacharomyces cerevisiae*. It is an essential component of the Rix1 complex (Rix1p/Ipi2p-Ipi1p-Ipi3p) that is required for the processing of 35S pre-rRNA in pre-60S ribosomal particles and for the initiation of DNA replication. The human IPI3 homolog is WDR18 (WD repeat domain 18), which shares significant homology with yIpi3p. Here we report that knockdown of hIPI3 resulted in substantial defects in the chromatin association of the MCM complex, DNA replication, cell cycle progression and cell proliferation. Importantly, hIPI3 silencing did not result in a reduction of the protein level of hCDC6, hMCM7, or the ectopically expressed GFP protein, indicating that protein synthesis was not defective in the same time frame of the DNA replication and cell cycle defects. Furthermore, the mRNA and protein levels of hIPI3 fluctuate in the cell cycle, with the highest levels from M phase to early G1 phase, similar to other pre-replicative (pre-RC) proteins. Moreover, hIPI3 interacts with other replication-initiation proteins, co-localizes with hMCM7 in the nucleus, and is important for the nuclear localization of hMCM7. We also found that hIPI3 preferentially binds to the origins of DNA replication including those at the c-Myc, Lamin-B2 and β-Globin loci. These results indicate that hIPI3 is involved in human DNA replication licensing independent of its role in ribosome biogenesis.

## Introduction

In eukaryotes, the cell cycle can be divided in two major stages: the first is interphase, which is for cell growth, genome duplication and preparation for mitosis, and the second is mitotic phase in which the cell separates itself into two cells. Before a cell divides, it must replicate its genomic DNA so that the two daughter cells have the same genetic information as their parent.

The replicators and origins of replication are special sequences in the genome, serving as the binding sites for replication-initiation proteins and the start sites, respectively, for the initiation of DNA replication [1, 2]. The replication origins are usually AT rich sequences which are more easily unwound [3].

From late M to early G1 phase of the cell cycle, a pre-replicative complex (pre-RC) is assembled step-wise onto each replicator. Formation of the pre-RC, also referred to as replication licensing, is required for DNA replication initiation. The pre-RC is composed of six ORC proteins (Orc1-6p), Noc3p, Cdc6p, Cdt1p, and a heterohexamer of MCM proteins (Mcm2-7p) [1–3]. In addition, Ipi1-3p has also been shown to be part of the pre-RC in budding yeast [4]. The pre-RC then helps to recruit other initiation proteins to form the pre-initiation complex (pre-IC) in late G1 phase. At the G1-to-S transition, cycle-regulated phosphorylation of Orc2p, Orc6p, Cdc6p, and MCM proteins by the cyclin-dependent protein kinase (CDK) and the Dbf4p-dependent Cdc7p kinase (DDK) activates replication and also blocks re-initiation within the same cell cycle [5]. After one round of DNA replication, the phosphatase Cdc14p dephosphorylates ORC, Cdc6p and MCM proteins to reset the replication origins to the competent state for the next pre-RC assembly at the M1-to-G1 transition [6].

An IPI (involved in processing ITS2) complex consists of Ipi1p, Ipi2p, and Ipi3p was first identified in budding yeast [7]. *S*. *cerevisiae* Ipi1-3p have been found to be required for cell viability and for processing of the ITS2 sequences from 35S pre-rRNA in pre-60S ribosomal particles [8, 9]. Ipi3p also functions as a component of the Five Friends of Methylated CHTOP (5FMC) complex which is recruited to ZNF148 by methylated CHTOP, leading to desumoylation of ZNF148 and subsequent transactivation of ZNF148 target genes [10]. Human IPI3 was predicted to play a possible role in the assembly of the large ribosomal subunit in a computational analysis of large-scale protein-protein interactions [11].

We have reported the function of Ipi3p in DNA replication licensing in budding yeast [4]. Ipi3p interacts with other pre-RC proteins and replication origins and is required for pre-RC assembly and maintenance independent of its function in ribosome biogenesis in budding yeast [4]. The human homolog of yIpi3p is WD repeat domain 18, which is a member of the WD repeat protein family, and hIPI3 protein shares significant homology with yIpi3p, with 22% identity and 40% similarity. One of the most important sequence homology between yIpi3p and hIPI3 is the WD40 repeat domain. The WD40 repeat domain is a structure of about 40 amino acids, and it usually locates at the end of a tryptophan-aspartic acid (WD) dipeptide. It was reported that a human protein containing five WD40 repeat domains can stabilize ORC binding to chromatin by interacting with ORC and modulating the chromatin association of ORC in human cells [12], suggesting that WD40 repeat proteins play important roles in human DNA replication. Here we show that hIPI3 also has important functions in the initiation of DNA replication in human cells.

## Materials and Methods

### Mammalian cell culture and harvest

HeLa, HEK 293T and A549 cells [13, 14] were cultured in Dulbecco’s Modified Eagles Medium (pH7.4) with 10% (v/v) Fetal Bovine Serum at 37°C in atmosphere of 5% CO_2_ and 100% humidity. Cells were harvested by incubation in 0.25% trypsin-EDTA (Gibco) at 37°C for 2 min followed by the addition of ice-cold PBS and centrifugation for 5 min at 3500 rpm. The harvested cells were washed with ice-cold PBS and collected by centrifugation at 3500 rpm for 5 min. For chromatin binding and immunoblotting assays, the cells were frozen in -80°C before use. For FACS, the cells were resuspended in PBS and fixed in 70% ethanol at -20°C.

### Cell transfection with siRNA

The cells were transfected with different siRNAs (Table 1) using Lipofectamine RNAiMAX (Invitrogen) according to the manufacturer's instructions. The final concentration of siRNA in the cell culture was 20 nM unless noted otherwise. After the first transfection, the cells were cultured for 24 hrs, and the second transfection was performed in the same way as the first transfection [15]. The transfected cells were used for assays 48 hrs post the second transfection.

**Table 1:** siRNAs for hIPI3.

Name	Sense (5'-3')	Antisense (5'-3')
IPI3si#1	CUCCUGuCUUCAGUUCAuAdTdT	UGUGAACUGAAGGCAGGAGdTdT
IPI3si#2	ACCUCCUuACUGGACuAGAdTdT	UCUGGUCCAGUGAGGAGGUdTdT
IPI3si#3	GGACUCUCuUCAGUUCUGUdTdT	ACAGAACUGAGGAGAGUCCdTdT
IPI3si#4	CCUCCUuACUGGACuAGAudTdT	GUCUGGUCCAGUGAGGAGGdTdT
IPI3si#5	AGAUCAAUuGGGACuUGUUdTdT	AACAGGUCCCGAUUGAUCUdTdT
IPI3si#6	UCAGUUCUGUGUCGUGUUudTdT	GAACACGACACAGAACUGAdTdT
NC	CTCTTAGCCAATATTCGCTdTdT	AGCGAATATTGGCTAAGAGdTdT

Note: The u residues were introduced to replace the C residues on the sense strain to increase silencing efficiency [16]; NC, negative control siRNA.

### Immunoblotting

Each protein sample was mixed well with the same volume of 2X Laemmli’s buffer, boiled at 100°C for 3–5 min, and then loaded onto 10 or 12.5% SDS-PAGE gels. The proteins were then transferred onto nitrocellulose membrane. TBST containing 5% dry milk was used to block the membrane for 30 min, and the proteins on the membrane were probed with specific primary antibodies: Anti-WDR18 (anti-hIPI3; Proteintech, 15165-1-AP, 1:800), Anti-MCM3 (Santa Cruz, sc-9850, 1:1,500), Anti-MCM4 (Santa Cruz, sc-9840, 1:1,500), Anti-MCM6 (Santa Cruz, sc-9845, 1:1,500), Anti-ORC2 (Santa Cruz, sc-32734, 1:1,500), Anti-ORC3 (Santa Cruz, sc-21862, 1:2,000), Anti-ORC4 (Santa Cruz, sc-19725, 1:2,000), Anti-Histone (Santa Cruz, sc-11278, 1:2,000), anti-BM28 (Mcm2; Becton Dickinson, 610701, 1:1,500), anti-Mcm7 (Santa Cruz, sc-9966, 1:1,000), anti-Cdc6 (Santa Cruz, sc-9964, 1:1,500), anti-Orc6 (Santa Cruz, sc-32735, 1:1,500), anti-β-actin (Sigma, A2228, 1:1,000), or anti-HA (Roche, 12CA5, 1:20,000). The signals were visualized using the SuperSignal^™^ reagent (Pierce) after secondary antibodies bound to the primary antibodies on the membrane.

### Chromatin binding assay

Cells were grown in 6-well plates and treated as needed. Cells were harvested and resuspended in two cell-pellet volumes of extraction buffer (EB; [17]) with vortexing and setting on ice for 10 min. Some 20 ul of 30% ice-cold sucrose containing protease inhibitors were added to the bottom of the tube. The tube was centrifuged at 15,000 rpm for 10 min at 4°C. The supernatant was transferred to a new tube. The pellet was washed with 20 ul of EB. The supernatants from the last two steps were combined and saved as the supernatant fraction, and the pellet was saved as the chromatin fraction. The pellet was resuspended in 30 μl of EB, mixed well by vortexing, added with 30 μl of 2X Laemmli’s buffer, mixed well by vortexing, and boiled for 3 min. Some 60 μl of 2X Laemmli’s buffer were added to the supernatant, and the contents were mixed by vortexing and boiled for 3 min. After boiling, the tube was cooled on ice for 1 min, mixed well, and centrifuged at 15,000 rpm for 5 min. The protein samples were loaded onto SDS-PAGE gels at room temperature.

### EdU incorporation assay

Some 24 hrs after the second transfection with siRNA, the cells were treated with Mimosine for 22 hrs (G1/S boundary). The cells were released from the Mimosine block by washing with DMEM three times. Two hrs later, EdU was added at a final concentration of 100 μM. After 30 min of incubation at 37°C, cells were fixed with 4% paraformaldehyde in PBS for 30 min at room temperature. The cells were washed with PBS three times and then treated with 0.5% Triton X-100 in PBS for 10 min. The samples were stained with Apollo for 60 min at room temperature, washed with methanol for 5 min twice and then with PBS for 5 min twice. Cells were photographed under a fluorescence microscope (Nikon TE2000E), and EdU incorporation was observed for at least 300 cells per sample. All values were means of three independent experiments.

### FACS analysis for mammalian cells

Cells were fixed with 70% ethanol and centrifuged at 7500 rpm for 10 min at 4°C. After washing with PBS, the cells were stained with DNA staining reagent propidium iodide (20 ug/ml) at 4°C for 1 hr. At least 10,000 cells per sample were analyzed with a flow cytometer (BD).

### Real-time quantitative reverse transcription-PCR (qRT-PCR)

Total RNA was isolated using TRIzol reagent (Invitrogen). RNA was reverse transcribed with First Strand cDNA Synthesis Kit (Fermentas) according to the manufacturer’s instructions. The cDNA samples were stored at -20°C until use. Quantitative PCR was performed with the Applied Biosystems 7500 Fast Real-Time PCR System using FastStart Universal SYBR Green Master (Rox) kit (Roche). The primers used were listed on Table 2. The gene expression level of hIPI3 was normalized to the internal control glyceraldehyde 3-phosphate dehydrogenase (GAPDH) and was expressed in percentage relative to the untreated cells.

**Table 2:** Primers for hIPI3 qRT-PCR.

Name	Sequence (5'-3')
hIPI3#1F	CTCAATGGCGAGTATCTG
hIPI3#1R	CATGATCTTCTGCTGGAG
GAPDH#2F	CCCTCAACGACCACTTTGTC
GAPDH#2R	AGGGGAGATTCAGTGTGGTG

### Coimmunoprecipitation assay

Some 24 hrs after transfection with plasmid DNA expressing HA-tagged hIPI3, HEK 293T cells in one 10-cm dish were lysed by incubation on ice for 10 min in 400 μl of Lysis/IP buffer containing protease inhibitors [18] followed by cell scraping off the plate. The lysate was transferred into a 1.5-ml Eppendorf tube, and the dish was washed with 400 ul of Lysis/IP buffer. Total protein extracts were collected after centrifugation at 10,000 rpm for 10 min at 4°C. Some 50 ul of total protein extracts were kept as whole cell proteins, and the remaining extracts were split into two tubes: one for IP and the other for mock-IP with control IgG. Protein extracts were pre-cleared with 50 ul of protein G beads for each IP by rotating the tube for 1 hr at 4°C. The protein G beads were removed by centrifugation at 10,000 rpm for 10 min at 4°C. Five ug of anti-HA antibody or control mouse IgG antibody were added to each IP or mock-IP tube, and the tubes were rotated overnight at 4°C. Fifty ul of protein G beads were added to each tube, and the tubes were rotated for 2 hrs at 4°C. The tubes were centrifuged at 3,000 rpm for 5 min at 4°C. The supernatants were removed, and each pellet was washed four times with 1 ml of Lysis/IP buffer and collected by centrifugation at 3,000 rpm for 5 min at 4°C. After the last centrifugation, the pellets were centrifuged at 10,000 rpm for 2 min, and the residual supernatants were carefully removed. A desired volume of 2X Lammili’s buffer was added to each pellet, and the samples were boiled at 95°C for 5 min before being used for immunoblotting.

### Cell synchronization

Cell culture at 40% confluency was added with DMEM (10% FBS) containing 2 mM Mimosine and incubated for 22 hrs (G1/S boundary block). After the Mimosine arrest, Mimosine was removed by washing with fresh DMEM three times, each with incubation for 2 min at 37°C. After release from Mimosine for 8 hrs, 100 ng/ml Nocodazole was added to the medium for 12 hrs (mitotic block). Nocodazole was then removed by washing with fresh DMEM to release the cells.

### Chromatin immunoprecipitation (ChIP) assay

ChIP assays were performed using a published protocol [19] with modifications. In brief, approximately 2x10^7^ HEK-293T cells transiently expressing HA-hHPI3 or HeLa cells expressing stably tagged HA-hIPI3 were incubated for 15 min in DMEM containing 1% formaldehyde at room temperature. The cross-linking was then quenched by adding glycine to 125 mM. Cells were washed with ice-cold PBS three times, scratched off, and then transferred to Eppendorf tubes. Cells then were lysed using SDS lyses buffer containing proteinase inhibitors. The lysed cells was sonicated to shear chromosomal DNA to 200–500 bp. The samples were then diluted 10 times in IP dilution buffer and incubated overnight at 4°C with 5 ng of antibody each. Some 50 ul of 50% Protein-G beads were then added to each, and the tube was incubated by rotating overnight at 4°C. The immunoprecipitates were washed sequentially with low salt buffer, high salt buffer, IP wash buffer and lyses buffer. After the last wash, the samples were eluted by elution buffer. Cross-linking was reversed by incubation in 0.3 M NaCl overnight at 65°C. The samples were then treated with Proteinase K (40 ug/ml) at 45°C for 2 hrs, and the DNA was purified by extraction with 25:24:1 phenol/chloroform/isoamyl alcohol. The amount of the precipitated DNA was analyzed by RT-PCR with different primers (Table 3).

**Table 3:** Primers for Detecting Replication Origins and the Control Sequences.

Name	Sequence
Myc-ori-F	TACAGACTGGCAGAGAGCAG
Myc-ori-R	ATGTATGCACAGCTATCTGG
Myc-con-F	GGTTCTAAGATGCTTCCTGG
Myc-con-R	TGGTTGTGAAGGCAGCAGAA
LMNB2-ori-F	GCGTCACAGCACAACCTGC
LMNB2-ori-R	GAGGCAGAACCTAAAATCAAA
LMNB2-con-F	GCTGCGCTCAGGTTAAGAAG
LMNB2-con-R	GTGCTCACGGCAGATAAGGT
β-globin-ori-F	GGTGAAGGCTCATGGCAAGA
β-globin-ori-R	AAAGGTGCCCTTGAGGTTGTC
β-globin-con-F	GGCAAGAAGGTGCTGACTTCC
β-globin-con-R	GCAAAGGTGCCCTTGAGATC

### Colony formation assay

Colony formation assays were performed according to a published method [20] with modifications. Some 72 hrs after different treatments, HeLa cells were digested with trypsin to produce single-cell suspension. About 1x10^3^ cells were seeded to 6-well plates. The cells were cultured with DMEM (10% FBS) for three weeks. The medium was removed from the plates, and the cells were washed with PBS. The cells were then fixed using 4% paraformaldehyde in PBS containing 0.5% crystal violet and incubated in staining buffer for 30 min at room temperature. The staining buffer was removed and the plates were washed with PBS. Each well was photographed, and the numbers of the colonies were counted using ImageJ [21].

### Immunofluorescence assay

Cells were seeded on sterile glass cover slips on 12- or 24-well plates, washed with PBS and fixed for 15 min with 4% paraformaldehyde solution (pH7.4). The cells were permeablized by incubating for 15 min on ice with 0.1% Triton-X-100 in PBS. The cells were washed 5 times with PBS and incubated for 1 hr with 1% BSA (w/v) in PBS. Primary antibodies diluted 1:50 in blocking buffer were added to the cover slips which were then incubated at 4°C overnight. The cells were washed 5 times with PBS and incubated in secondary antibodies (1:500 in blocking buffer) in a dark humidity chamber at 4°C for 2 hrs. At last, the cells were washed 5 times with PBS. The cover slips were mounted by pressing them onto glass slides. Photos of cells were taken with a fluorescence microscope (Nikon TE-2000).

### Yeast two-hybrid Assay

The ORFs of DNA replication-initiation proteins (hORC1-6, hMCM2-7 and hIPI3) were generated by PCR and cloned into the pGADT7 and pGBKT7 vectors. Yeast two-hybrid analysis was performed as previously described [22, 23].

## Results

### Silencing of hIPI3 resulted in substantial defects in the chromatin association of MCM proteins, DNA replication, cell cycle progression and cell proliferation in human cells

In budding yeast, Ipi3p has been showed to be required for replication licensing [4]. To determine if the human IPI3 also plays important roles in DNA replication, we designed several siRNAs [Tables 1 and 2] to knock down hIPI3. The qRT-PCR results showed that the siRNA#3–6 were active in silencing hIPI3; #6 being the most active one, with silencing by over 80% at 20–40 nM at both the mRNA and protein levels (S1 Fig). The chromatin binding assay results also demonstrated that most of hIPI3 protein bound to the chromatin (S1B Fig).

**Fig 1:**
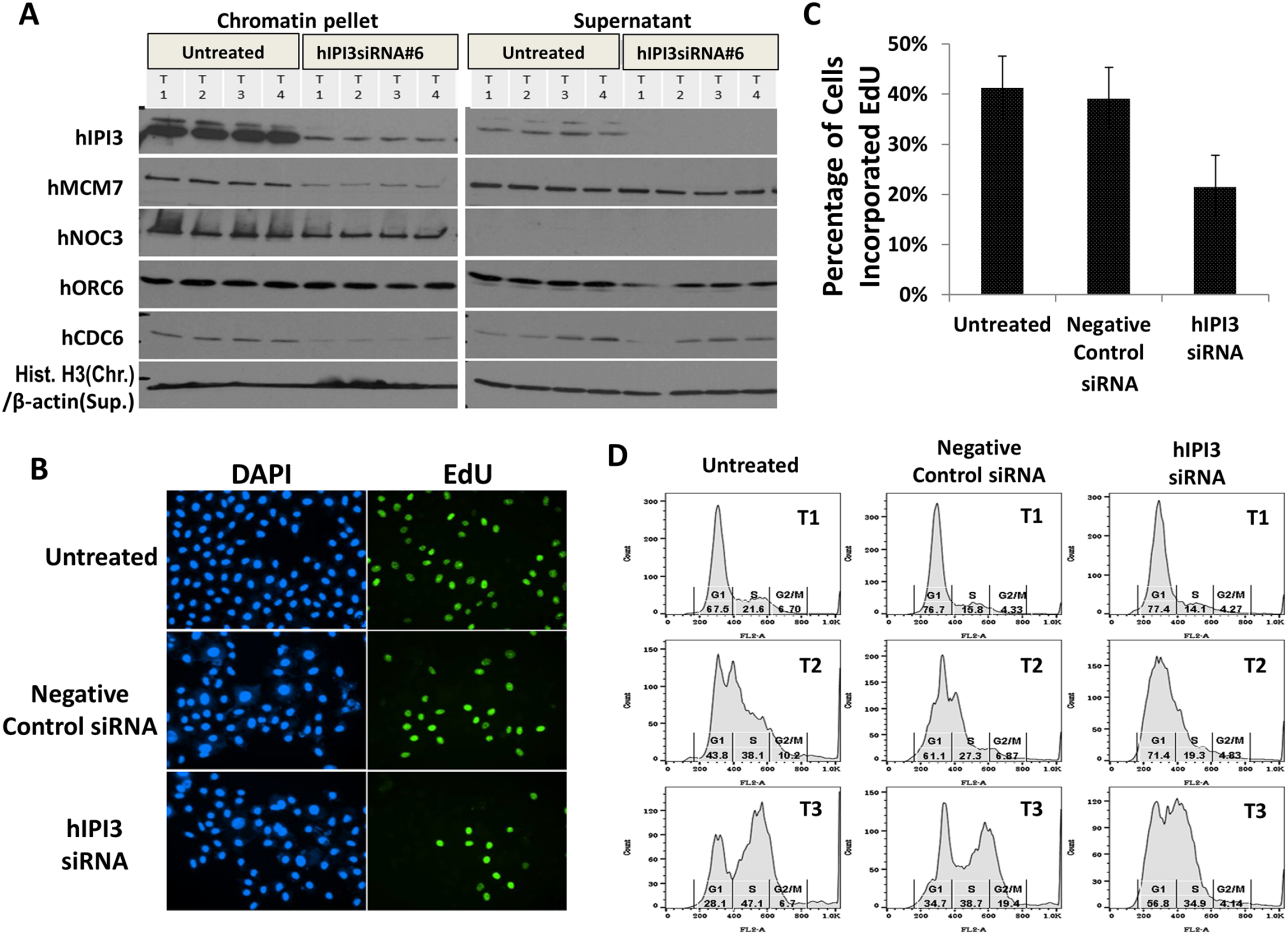
The hMCM7 and hCDC6 levels on chromatin were significantly reduced and DNA replication and cell cycle progression were substantially defective after hIPI3 silencing. (A) HeLa cells were treated with hIPI3 siRNA, blocked at the G1/S boundary by Mimosine (T1), released and then harvested at different time points (T2–T4 for 3, 6 and 9 hrs post release, respectively) for chromatin-binding assays to detect the pre-RC proteins as indicated on the chromatin and supernatant fractions. Untreated cells were used as the control. Histone H3 and β-actin were used as the loading controls for the chromatin and supernatant fractions, respectively. The results show that knockdown of hIPI3 resulted in defective chromatin association of hMCM7 and hCDC6, but not of hNOC3 or hORC6. (B-D) HeLa cells were treated and harvested for EdU incorporation assay (B and C) and flow cytometry (D) in a similar way to that described in (A). The EdU incorporation assay results at 2 hrs post release show that DNA replication was partially defective and the flow cytometry data at T1–T3 corresponding to the time points in (A) indicate that cell cycle progression slowed down after hIPI3 silencing.

During the cell cycle, Orc1-6p and Noc3p are present at replication origins across the cell cycle, and other pre-RC components including Ipi3p, Cdc6p, Mcm2-7p and Cdt1p bind to chromatin during the M-to-G1 transition [1–3]. In budding yeast, Ipi3p is required for the loading and maintenance of MCM proteins on chromatin [4]. We performed chromatin-binding assay to determine whether or not hIPI3 also has this function. HeLa cells were treated with hIPI3 siRNA and then blocked at the G1/S boundary by Mimosine, released and harvested at 2, 4 and 6 hrs after release. The chromatin-binding assay results showed that knockdown of hIPI3 resulted in substantial defects in the chromatin association of hMCM7 and hCDC6, but not of hNOC3 or hORC6, in Mimosine-arrested cells (Fig 1A; T1) and those after release from Mimosine (Fig 1A; T2–T4). Since all pre-RC proteins are normally present on chromatin at the G1/S boundary in untreated cells without hIPI3 silencing, these results suggest that hIPI3 is involved in replication licensing upstream of hCDC6 and MCM proteins and downstream of hORC and hNOC3, reminiscent of the findings in budding yeast [4].

We then examined if hIPI3 is also important for DNA replication and cell cycle progression using EdU incorporation assay and flow cytometry, respectively. HeLa cells after hIPI3 silencing were arrested at the G1/S boundary with Mimosine, released into S phase and then harvested. The EdU incorporation assay results showed that the percentage of cells incorporated EdU was reduced by ~50% after hIPI3 silencing compared to untreated cells at 2 hrs post release (Fig 1B and 1C). The flow cytometry data indicated that cell cycle progression was noticeably defective after hIPI3 silencing, as hIPI3-silenced cells had more G1 phase cells and fewer S phase cells compared to the untreated and control siRNA-treated cells at both 3 and 6 hrs post release (Fig 1D). These data strongly suggest that hIPI3 is important for DNA replication and cell cycle progression in human cells. The reason why DNA replication and cell cycle progression were only partially defective was probably because silencing of hIPI3 was incomplete and thus some replication origins were licensed (Fig 1A). It's well known that partial DNA replication and cell cycle progression can occur when only a small number of replication origins are activated.

**Fig 2:**
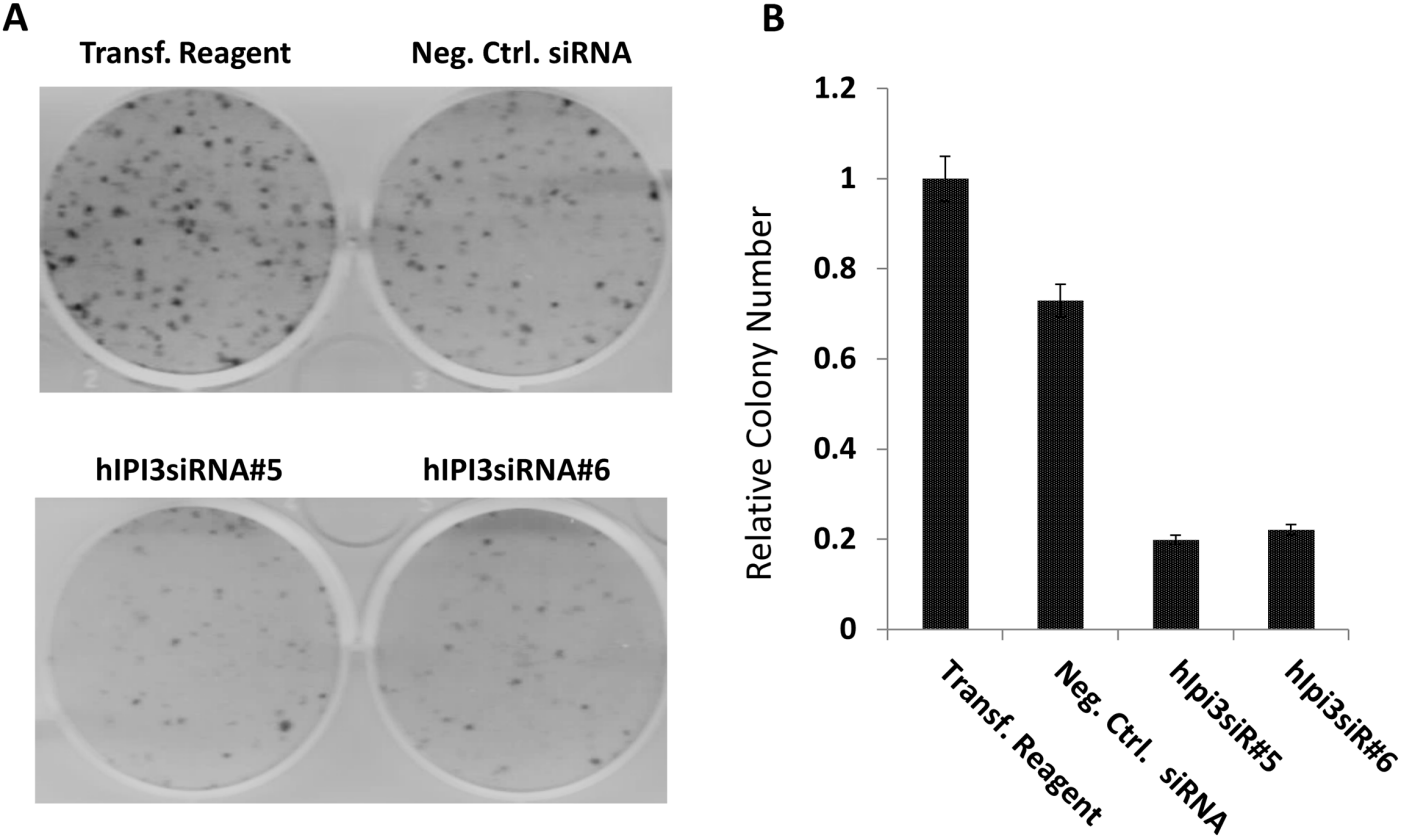
Cell proliferation was largely inhibited after hIPI3 silencing. HeLa cells were treated with the transfection reagent, negative control siRNA or two different hIPI3 siRNAs and grown for 3 weeks for the colony formation assay. The numbers of colonies were normalized to that of the transfection agent-treated cells. The data show that after siRNA silencing, the number of colonies was significantly lower that the two controls.

DNA replication is prerequisite for cell proliferation. We performed colony formation assay to demonstrate that cell proliferation was indeed inhibited after hIPI3 silencing. HeLa cells were treated with siRNA transfection reagents, negative control siRNA or hIPI3 siRNA and grown for 3 weeks. Comparing to the transfection reagent-treated cells and negative control siRNA-treated cells, both the colony number and colony size of the hIPI3 siRNA-treated cells were significantly reduced (Fig 2), indicating that hIPI3 silencing resulted in defective cell proliferation.

**Fig 3:**
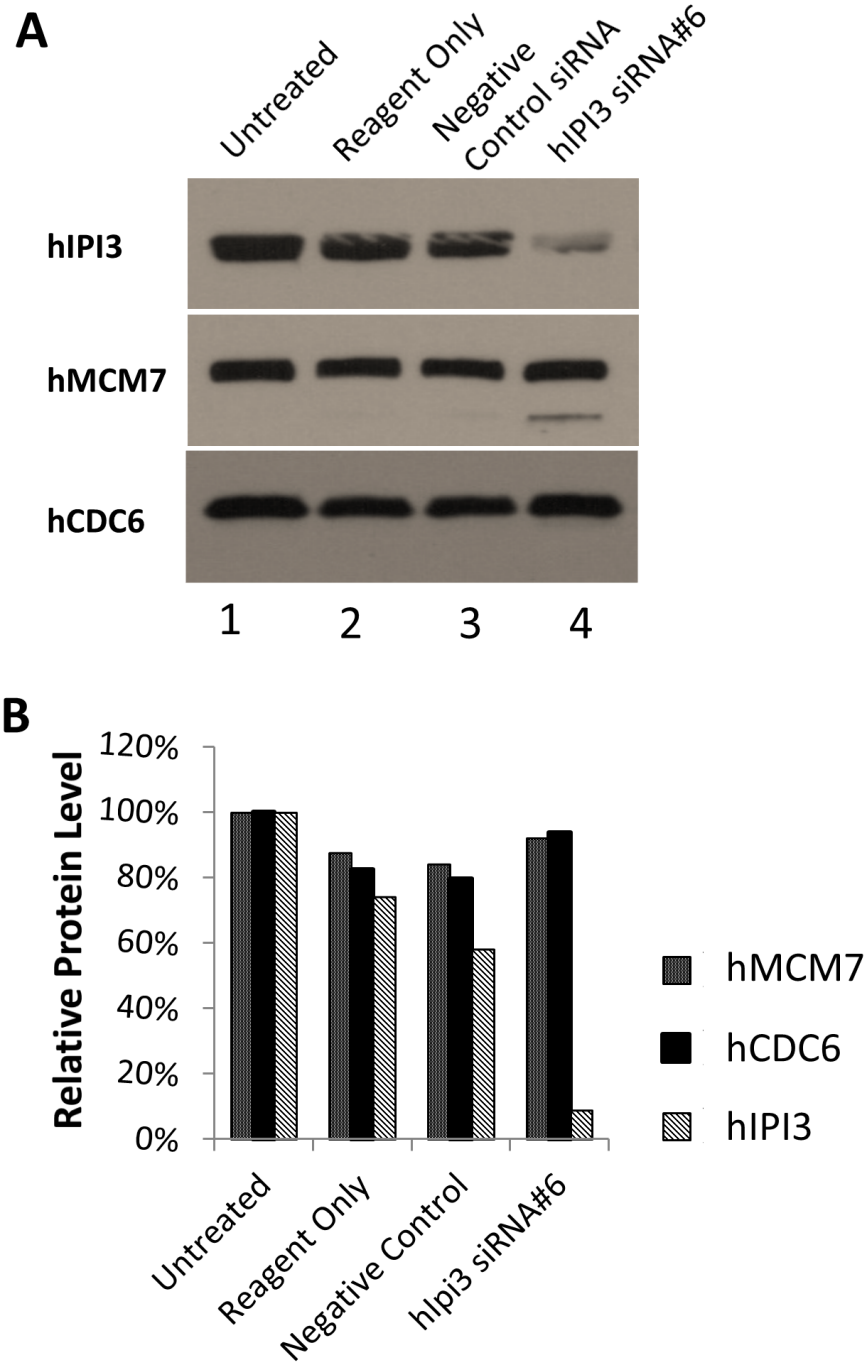
The hMCM7 and hCDC6 total protein levels were not reduced after hIPI3 silencing. HeLa cells were treated with hIPI3 siRNA, and the protein levels of hIPI3, hMCM7 and hCDC6 were analyzed by immunoblotting (A), and relative signal intensities were quantified (B). The results demonstrate that even though the hIPI3 protein has been knocked down by ~90%, the hMCM7 and hORC6 protein levels were not reduced.

**Fig 4:**
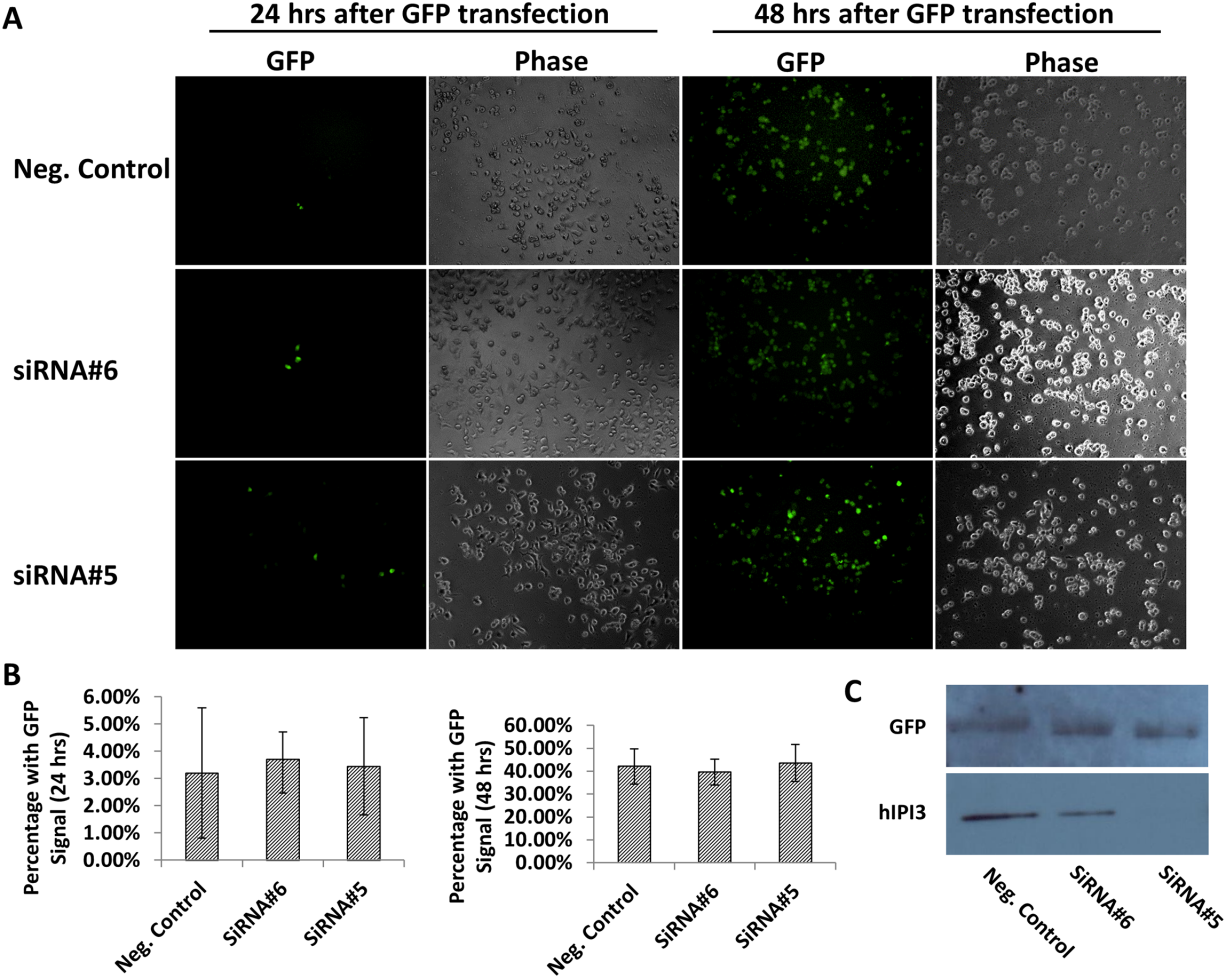
Ectopic GFP expression was not affected after hIPI3 silencing in the same time frame when the pre-RC defects were found. HeLa cells were transfected with GFP-expressing plasmid after hIPI3 siRNA silencing. (A) The immunofluorescence results indicate that at 24 and 48 hrs after the hIPI3-silenced cells were transfected with the GFP-expressing plasmid, the percentage of GFP-positive cells or the GFP fluorescence intensity was not reduced in cells treated with hIPI3 siRNA compared to those treated with the negative control siRNA. (B) Quantification of the percentage of cells with GFP signals from multiple images from (A). (C) The immunoblotting results show that the protein level of GFP at 48 hrs post-transfection with the GFP-expressing plasmid was not reduced after hIPI3 silencing.

Because hIPI3 also has an important function in ribosome biogenesis, we examined by immunoblotting whether or not hIPI3 silencing resulted in a reduction of the total protein levels of MCM proteins and/or hCDC6. The results showed that when the hIPI3 protein had been knocked down by ~90%, the cellular levels of hMCM7 and hCDC6 were not reduced (Fig 3A and 3B). Furthermore, when we transfected hIPI3-silenced cells with a GFP-expressing plasmid, the ectopic expression of GFP was not reduced judging by the percentage of GFP-positive cells and GFP fluorescence identity at 24 and 48 hrs post-transfection with the plasmid (Fig 4A and 4B) and also by immunoblotting at 48 hrs post-transfection with the plasmid (Fig 4C), suggesting that protein synthesis was little affected in the same time frame after hIPI3 silencing when defective pre-RC formation, DNA replication and cell cycle progress were found (Fig 1). This is consistent with previous studies by others showing that the levels of ribosome and protein synthesis in the cells remained unchanged for quite some time after inhibition of ribosome biogenesis in yeast [24]. Taken together, these results strongly suggest that hIPI3 is required for DNA replication licensing independent of its function in ribosome biogenesis in human cells.

It is known that the mRNA and protein levels of many pre-RC proteins fluctuate in the cell cycle [25]. The pre-RC is formed in late mitosis to G1 phase, and the protein levels of several pre-RC components are highest in late M to G1 phase [26, 27]. We determined the mRNA level of hIPI3 in the cell cycle by qRT-PCR in synchronized cell populations. HeLa cells were arrested with Mimosine, released into S phase and harvested every 2 hrs (S2A Fig; T1–T5). The cells were then arrested with Nocodazole in M phase, released into G1 phase and harvested every 2 hrs after release (S2A Fig; T6–T10). The qRT-PCR results showed that the mRNA level of hIPI3 fluctuated during the cell cycle; the highest level being from M phase to early G1 phase (S2B Fig). We also found by immunoblotting that the protein level hIPI3 is also cell cycle regulated (S2C and S2D Fig). The highest protein level of hIPI3 was in late M to early G1 phase, i.e., peaked slightly earlier than other pre-RC proteins (S2C and S2D Fig). These results are consistent with the time frame of the hIPI3 action in replication licensing during later M to early G1 phase.

### hIPI3 interacts with some hORC and hMCM subunits and is required for the nuclear localization of hMCM7

**Fig 5:**
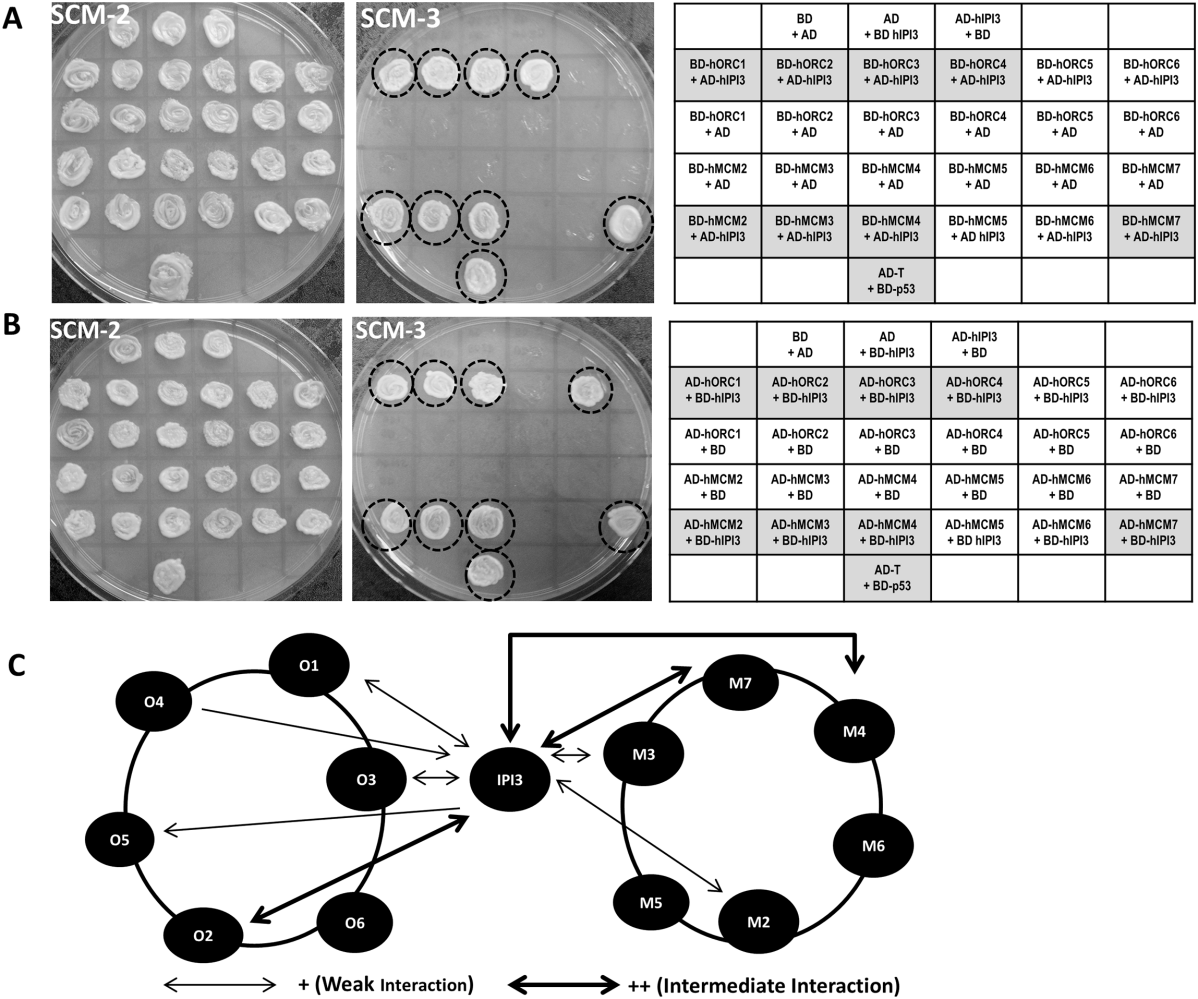
hIPI3 interacts with some hORC and hMCM subunits in the yeast two-hybrid system. AH109 yeast host cells were co-transformed with the indicated combinations of plasmids and grown on SCM-Trp-Leu (SCM-2) plates. (A, B) Transformants were then patched onto SCM-2 and SCM-Trp-Leu-His (SCM-3) plates and incubated, to test cell growth. Cells containing AD-T (SV40 large T-antigen) and BD-p53 were used as the positive interaction control. Cells containing individual plasmids and their opposite empty vectors were included as negative controls. Positive interactions are marked by circles in the photos and shades on the tables. (C) A summary diagram of the relative strength of the protein-protein interactions based on the data shown in (A, B) and S3A and S3B Fig. The symbols O1–O6 refer to hORC1-hORC6, and M2–M7 represent hMCM2-hMCM7. The thickness of the arrows indicates the observed relative strength of the interactions, with thin arrows representing weak interactions (recorded as “+” in S3B Fig) and the thicker arrows representing intermediate interactions (recorded as “++” in S3B Fig), relative to the strong interaction of the positive control (“+++”). Double arrows represent interactions detected in both directions, while single arrows indicate interactions detected in only one direction (eg. BD-hIPI3 interacts with AD-hORC5).

In budding yeast, yIpi3p interacts with yOrc2p, yCdc6p, yCdt1p and yMcm7p [4]. We performed yeast two-hybrid analysis to examine the possible physical interactions of hIPI3 with hORC1-6 and hMCM2-7. We found that hIPI3 interacted with hORC1, hORC2, hORC3, hMCM2, hMCM3 and hMCM7 in both directions of the yeast two-hybrid vectors (Fig 5 and S3 Fig). hIPI3 also had single-directional interactions with hORC4 (AD-hIPI3 with BD-hORC4) and hORC5 (AD-hORC5 with BD-hIPI3) (Fig 5 and S3 Fig). These results suggest that hIPI3 interacts with several ORC and MCM components as in budding yeast [4, 28, 29], implying an integral role of hIPI3 in the human pre-RC architecture.

**Fig 6:**
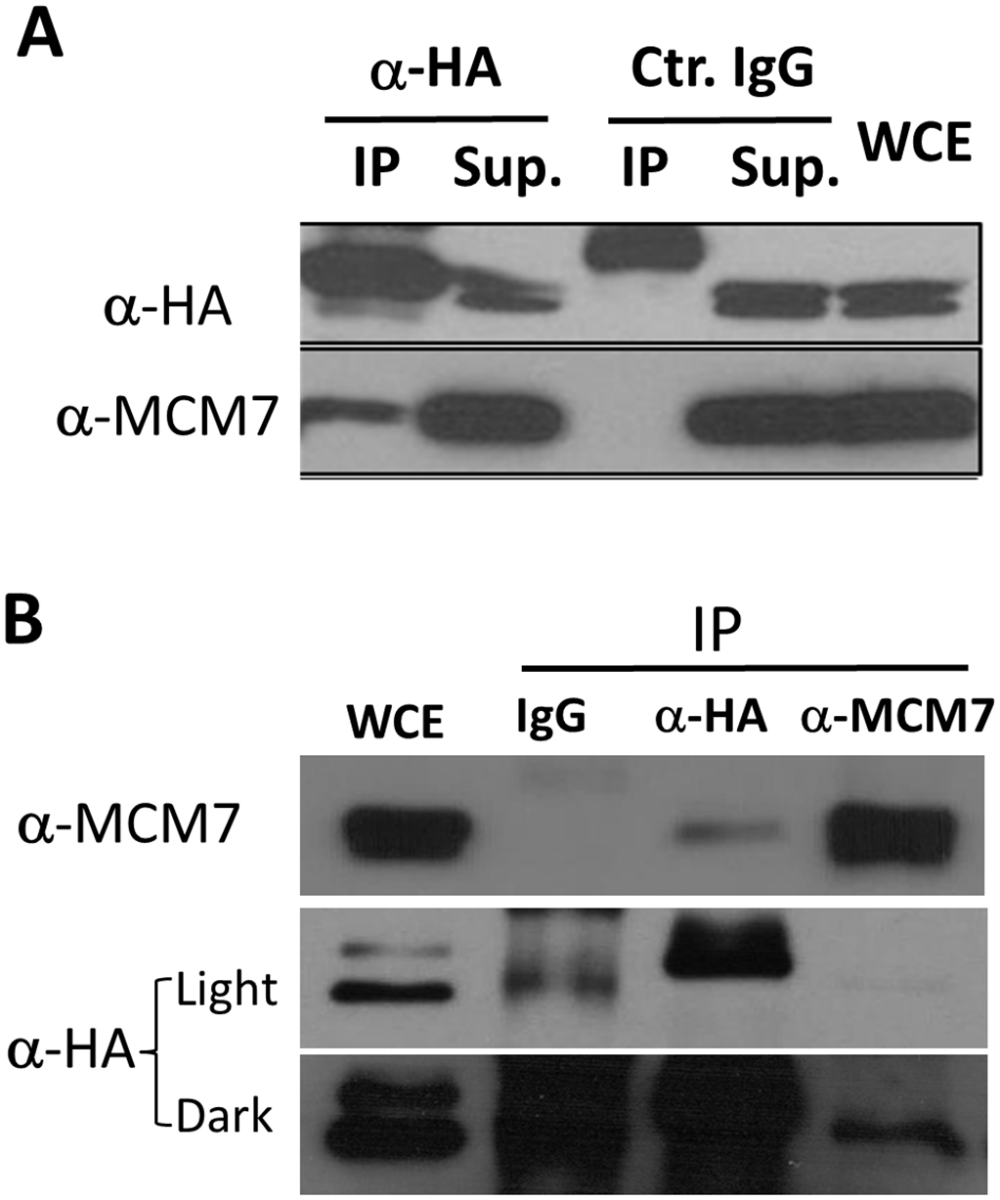
hIPI3 interacts with hMCM7 in co-IP from total cell extracts. (A) The HA-hIPI3 stable HeLa cells were arrested at the G1/S boundary by Mimosine, and whole-cell extracts (WCE) were immunoprecipated with an anti-HA antibody or the control mouse IgG. Co-IP assay results showed that the hIPI3 interacts with hMcm7. (B) An anti-Mcm7 antibody was used to perform a reverse co-IP experiment. hMCM7 was found to interact with HA-hIPI3 in the reciprocal co-IP.

We also employed co-immunoprecipitation (co-IP) to examine the interactions of hIPI3 with other pre-RC proteins in human cell extracts. The HA-hIPI3 stable HeLa-cells were arrested at the G1/S boundary by Mimosine, and whole-cell extracts (WCE) were immunoprecipitated with an anti-HA antibody. hMCM7 was found to be co-immunoprecipitated with HA-hIPI3 (Fig 6A). To confirm this interaction, an anti-hMCM7 antibody was used to perform a reverse co-IP experiment. The results indicated that hMCM7 could be co-immunoprecipitated with hIPI3 (Fig 6B). Currently we do not understand why the interactions between hIPI3 and some other pre-RC proteins found by yeast two-hybrid analysis were tested negative in co-IP (data not shown).

**Fig 7:**
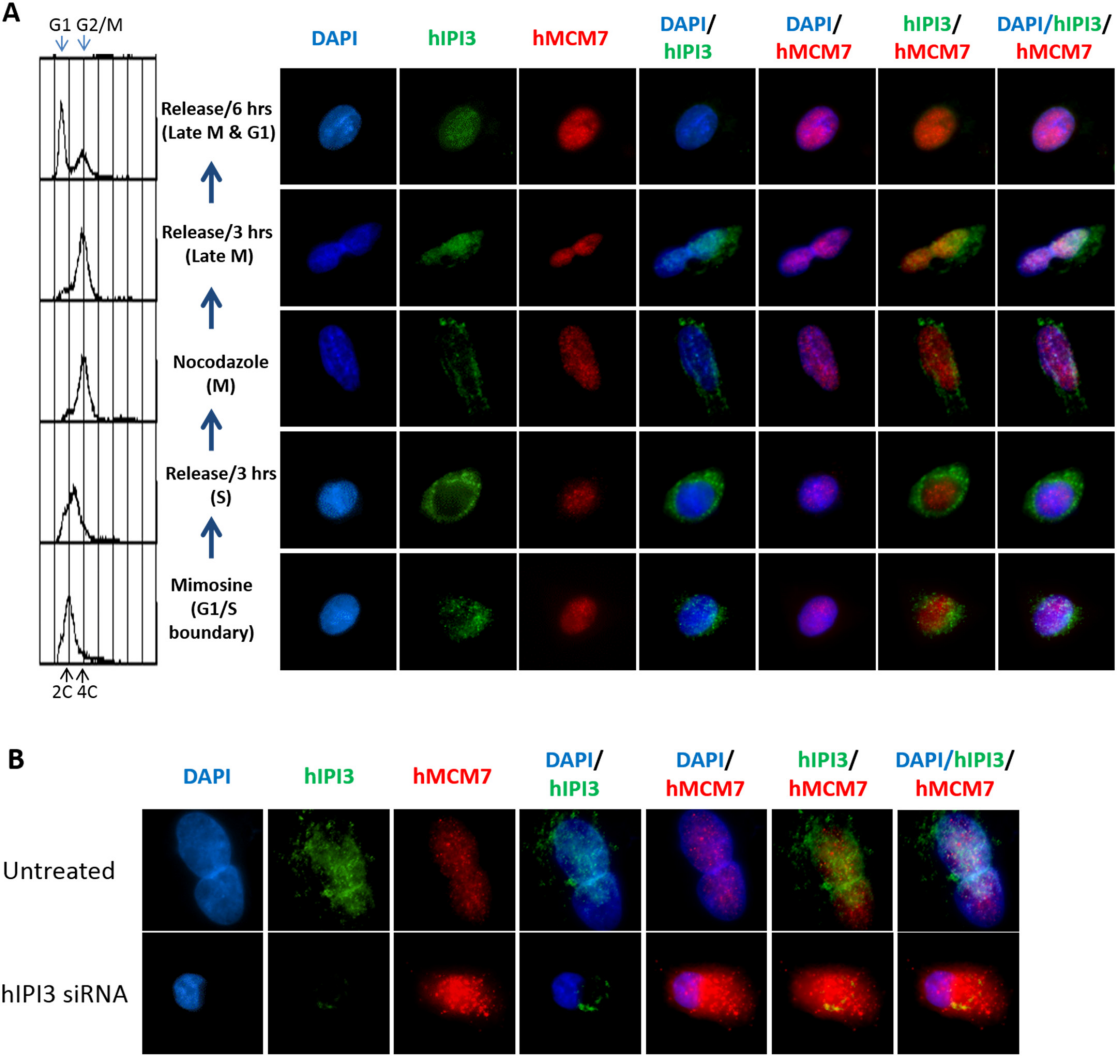
hIPI3 co-localizes with hMCM7 in the nucleus in late M and early G1 phases and hIPI3 is important for the nuclear localization of hMCM7. A549 cells were synchronized with Mimosine and then released for 3 hrs to obtain S phase cells. Afterwards, Nocodazole was used to arrest cells in M phase, and the cells were then released for 3 and hrs to obtain late M and late M & G1 cells, respectively. The cells were fixed with methanol and immuno-stained with anti-hIPI3 and anti-hMCM7, and the nuclear DNA was stained with DAPI. (A) Representative immunofluorescence images of the cells from each sample are shown here and in S4A Fig. The immunofluorescence results show that hIPI3 co-localized with hMCM7 in the nucleus in late M and G1 phases. (B) Asynchronous A549 cells with and without hIPI3 silencing were immune-stained with anti-hIPI3 and anti-hMCM7, and the nuclear DNA was stained with DAPI. Representative immunofluorescence images of the cells are shown here and in S4B Fig. The data show that after hIPI3 silencing, hMCM7 was largely absent from the nucleus in all cell cycle stages.

To further elaborate the interaction between hIPI3 and hMCM7, immunofluorescence microscopy was used to observe the localization of hIPI3 and hMCM7 during the cell cycle. A549 cells were arrested at the G1/S boundary by Mimosine and then released into the cell cycle. The cells were then arrested in M phase by Nocodazole and subsequently released into the next G1 phase. The results showed that hIPI3 localized in the cytoplasm at most of the time during the cell cycle, but co-localized with hMCM7 in the nucleus in late M and early G1 phase (Fig 7A and S4A Fig). Moreover, after hIPI3 silencing, most of hMCM7 was in the cytoplasm (only a small amount of hMCM7 overlapped with the DAPI signal in the nucleus) (Fig 7B and S4B Fig), suggesting that hIPI3 is important for the nuclear localization of hMCM7. Currently we do not understand the reason for this. It may be possible that failure in pre-RC formation can lead to disassembly, and thus nuclear exclusion of the MCM hexamers. It is interesting to note that when Cdt1p is depleted in yeast cells, the MCM complex also disassembles [30]. Together, these results further support the role of hIPI3 in pre-RC formation.

### hIPI3 binds to the replication origins at the c-Myc, β-globin and Lamin-B2 loci

**Fig 8:**
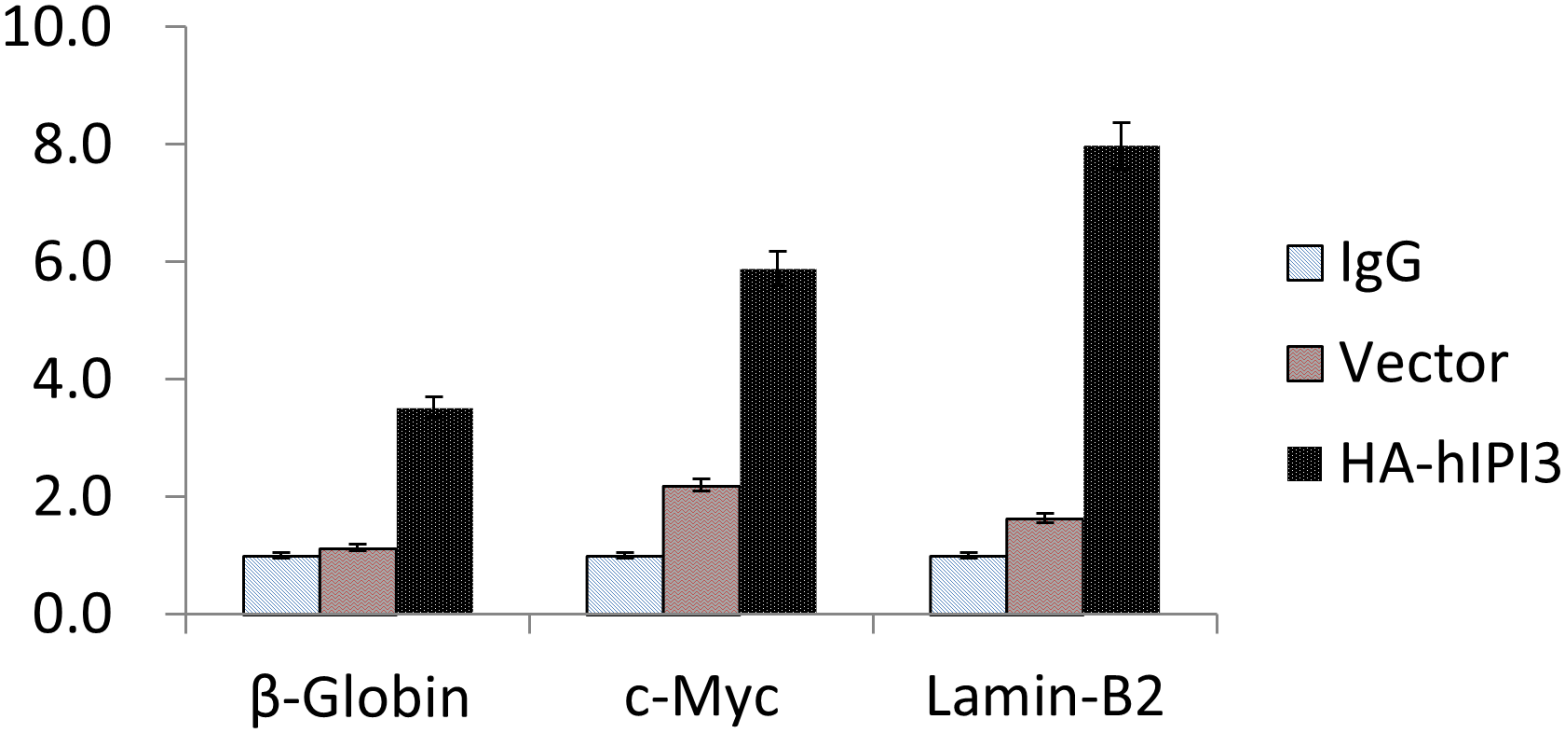
hIPI3 preferably binds to the origins of replication. HEK-293T cells were transfected with HA-hIPI3 expressing plasmid and harvested for ChIP assay with an anti-HA antibody. Mock ChIP with non-specific mouse IgG and anti-HA ChIP with extracts of the cells transfected with the empty vector were used as the negative controls. The relative amounts of the immunoprecipitated origin sequences and the non-origin control sequences were analyzed by qPCR, and the signals from the origin sequences were normalized to those of the non-original control sequences to obtain the relative levels, i.e., fold enrichment of the origin sequences compared to the control sequences). The ChIP assay results show that hIPI3 preferably bound to the origins of replication, including the β-globin, c-Myc and Lamin-B2 replication origins.

hMCM7 is known to bind to the origins of replication including the c-Myc and Lamin-B2 replication origins [31, 32]. We examined by chromatin immunoprecipitation (ChIP) if hIPI3 could also bind to these replication origins. HEK-293T cells were transfected with HA-hIPI3-expressing plasmid and harvested for ChIP assay with an anti-HA antibody. Mock-IP with non-specific mouse IgG and cells transfected with the empty vector were used as the negative controls. The relative amounts of the precipitated origin sequences were analyzed by qPCR and normalized to those of the non-original control sequences. The results showed that hIPI3 bound preferentially to the β-globin, c-Myc and Lamin-B2 replication origins, with 3.5-, 6- and 8-fold enrichment, respectively, of the origin sequences compared to the non-origin sequences (Fig 8). Similar data were also obtained with HeLa cells expressing stably tagged HA-hIPI3 (data not shown).

## Discussion

Previous studies have shown that the yeast Rix1 complex (Rix1-Ipi1-IPI3) is an essential component involved in ribosome biogenesis [4, 9] and DNA replication [4], and that yIpi3p plays an important role in the processing of 35S pre-rRNA in the pre-60S ribosomal particles [8]. hIPI3 belongs to the family of WD40 repeat domain-containing protein. The WD40 repeat domain-containing protein 1 (LRWD1) in human cells interacts with and co-localizes with ORC with similar dynamics as hORC during the cell cycle, serving to stabilize the chromatin association of ORC [12].

In our study, we show by hIPI3 silencing experiments that hIPI3 is required for human DNA replication licensing, cell cycle progression and cell proliferation. To further elucidate the role of hIPI3 in DNA replication, we utilized the yeast two-hybrid system to examine the physical interactions of hIPI3 with hORC1-6 and hMCM2-7. We found that hIPI3 interacts with several components of the human pre-RC complex, with the strongest interactions with hORC2 and hMCM7. Interestingly, this is consistent with previous studies showing that yIpi3 interacts with yOrc2p and yMcm7p [6]. We also confirmed by co-IP and immunostaining that hIPI3 interacts and co-localizes with hMCM7, and that their nuclear localization dynamics in the cell-cycle are similar. Moreover, we demonstrate that hIPI3 is important for the nuclear localization of hMCM7, and hMCM7 and hCDC6 cannot associate with the chromatin after hIPI3 silencing. These data strongly suggest that hIPI3 plays an important role in pre-RC assembly and maintenance. Consistent with this notion, we found that the mRNA and protein levels of hIPI3 fluctuate during the cell cycle as other pre-RC proteins do, with the highest mRNA and protein levels from late M phase to G1 phase which is the time window for pre-RC formation.

In yeast, the three IPI proteins bind to chromatin in an ORC- and Noc3p-dependent and cell cycle-regulated manner and bind preferably to replication origins [6]. Several origins of DNA replication in mammalian cells, including those at the β-globin, c-Myc origin and Lamin B loci, have been well characterized [33, 34]. The Lamin B origin has been mapped at the nucleotide resolution but several start sites and functional elements are observed for the c-Myc origin [35]. All MCM2–MCM7 subunits, CDT1, CDC6 and ORC2, are present in the c-Myc complex [36], and replication initiation at this origin occurs in early S-phase [37]. We found that hIPI3 binds to these three replication origins in human cells. The physical interactions of hIPI3 with other pre-RC proteins and with origins of DNA replication strongly suggest that hIPI3 plays a role in replication licensing directly at replication origins apart from its function in ribosome biogenesis which should not involve interactions with other pre-RC proteins or replication origins.

Previous studies by others suggest that the levels of ribosome and protein synthesis in the cells remained unchanged for quite a long time after inhibition of ribosome biogenesis by Pwp2p/Utp1p depletion in yeast [24]. Consistent with this, we show that the protein level of the endogenous hMCM7 and hCDC6 and the ecpotic GFP were mostly unchanged in the same time frame when pre-RC formation is defective after hIPI3 silencing, indicating that ribosome biogenesis defects could not be the cause of the pre-RC defects when we knocked down the hIPI3 in our experiments.

To summarize, we suggest that during late M phase to G1 phase of the cell cycle, hIPI3 binds to the upstream initiation proteins such as hORC and hNOC3 and serves as a loading factor to help to load the downstream pre-RC proteins including hCDC6 and hMCM7 proteins onto replication origins for replication licensing. As cells enter S phase, most hIPI3 localizes in the cytoplasm until the next late M to early G1 phase, helping to ensure that DNA replication occurs once and only once per cell cycle. Furthermore, in addition to being the actual components involved in both ribosome biogenesis and replication licensing, hIPI3 may also function as a regulator, mediator and/or substrate of the regulatory mechanisms that coordinate replication licensing and ribosome biogenesis to ensure the optimal resource utilization, fidelity of genome duplication and cell survival.

## Supporting Information

S1 Fig Silencing efficiencies of the siRNAs. (A) Six siRNAs were tested for their abilities to silence hIPI3 in HeLa cells. The mRNA level of hIPI3 quantified by qRT-PCR was reduced by ~80% using siRNA #3-#6 at 20–40 nM. (B) Chromatin-binding assays were performed with asynchronous HeLa cells (Untreated) or cells treated with the hIPI3siRNA#6 or the negative control siRNA. The results indicate that most of hIPI3 bound to the chromatin and that the protein level of hIPI3 bound to the chromatin was significantly reduced by hIPI3siRNA#6.
(TIF)

S2 Fig The hIPI3 mRNA and protein levels fluctuate during the cell cycle. (A) HeLa cells were arrested at the G1/S transition with Mimosine (T1), released into S phase and harvested every 2 hrs for 8 hrs (T2–T5). Afterwards, the cells were arrested in M phase with Nocodazole, released into G1 phase and then harvested every 2 hrs for 10 hrs (T6–T10). The cells were analyzed by flow cytometry for monitor cell cycle progression. (B) qRT-PCR results show that the mRNA level of hIPI3 fluctuated during the cell cycle and the highest level was between M phase and early G1 phase. (C) HeLa cells were arrested the G1/S transition with Mimosine (T1), released and harvested every 3 hrs for 6 hrs (T2 and T3). The cells were then arrested in M phase with Nocodazole, released and harvested every 2 hrs for 6 hrs (T4–T6) for immonoblotting. (D) Quantification of the immunoblotting data show that the highest protein level of hIPI3 was in late M to early G1 phase.
(TIF)

S3 Fig Additional data to show that hIPI3 interacts with some hORC and hMCM subunits in the yeast two-hybrid system. (A) AH109 cells transformants containing the indicated combinations of plasmids were streaked onto SCM-3 plates to examine the relative strength of the interactions. The combinations with positive interactions are marked by shades. (B) Summary of the results from (A). A single ‘+’ sign indicates a weak interaction while ‘++’ represents an intermediate interaction, relative to the strong interaction of the positive control which would be “+++”. Absence of the sign means no interaction.
(TIF)

S4 Fig More photos of the cells from the same experiments shown in Fig 7. Photos in S4A Fig show more cells from the same experiment as shown in Fig 7A, and Photos in S4B Fig show more cells from the same experiment as shown in Fig 7B.
(TIF)
