# Supplementary figures and images for: A Role of hIPI3 in DNA Replication Licensing in Human Cells
Source: PLoS One. 2016 Apr 8;11(4):e0151803. doi: 10.1371/journal.pone.0151803 (PMC4825987; doi:10.1371/journal.pone.0151803)

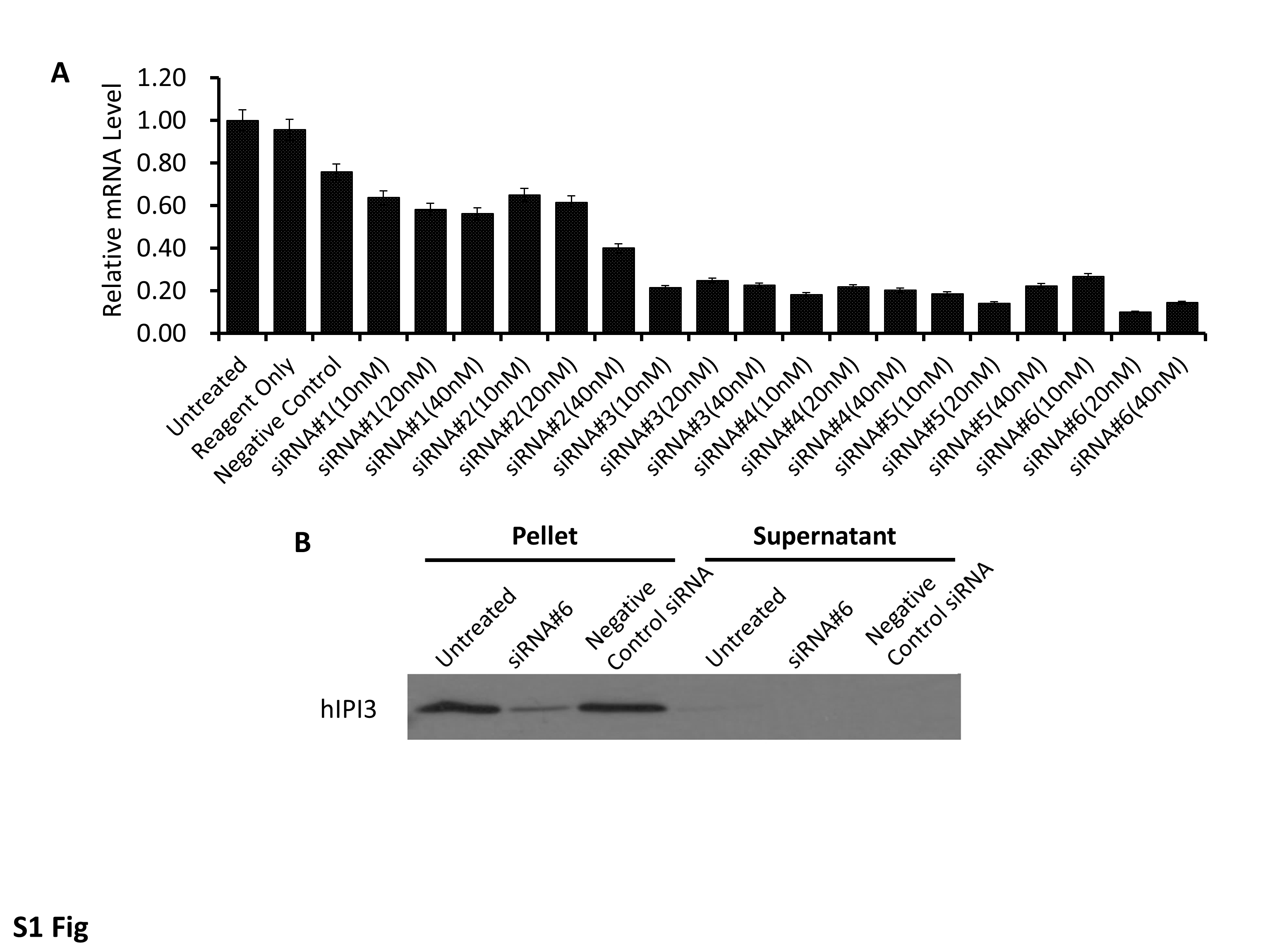

Supplement: S1 Fig — (A) Six siRNAs were tested for their abilities to silence hIPI3 in HeLa cells. The mRNA level of hIPI3 quantified by qRT-PCR was reduced by ~80% using siRNA #3-#6 at 20–40 nM. (B) Chromatin-binding assays were performed with asynchronous HeLa cells (Untreated) or cells treated with the hIPI3siRNA#6 or the negative control siRNA. The results indicate that most of hIPI3 bound to the chromatin and that the protein level of hIPI3 bound to the chromatin was significantly reduced by hIPI3siRNA#6. (TIF) [file pone.0151803.s001.TIF]

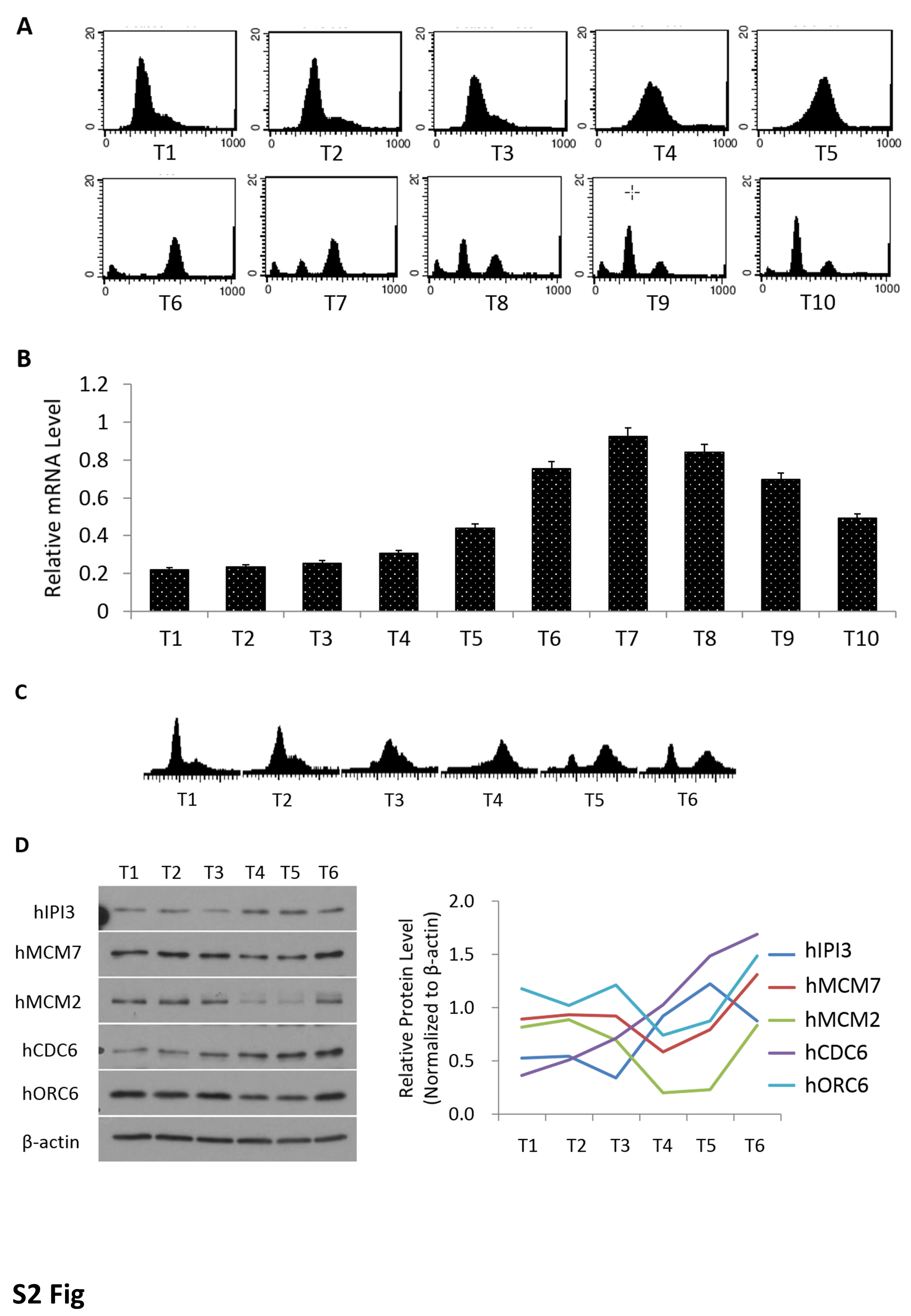

Supplement: S2 Fig — (A) HeLa cells were arrested at the G1/S transition with Mimosine (T1), released into S phase and harvested every 2 hrs for 8 hrs (T2–T5). Afterwards, the cells were arrested in M phase with Nocodazole, released into G1 phase and then harvested every 2 hrs for 10 hrs (T6–T10). The cells were analyzed by flow cytometry for monitor cell cycle progression. (B) qRT-PCR results show that the mRNA level of hIPI3 fluctuated during the cell cycle and the highest level was between M phase and early G1 phase. (C) HeLa cells were arrested the G1/S transition with Mimosine (T1), released and harvested every 3 hrs for 6 hrs (T2 and T3). The cells were then arrested in M phase with Nocodazole, released and harvested every 2 hrs for 6 hrs (T4–T6) for immonoblotting. (D) Quantification of the immunoblotting data show that the highest protein level of hIPI3 was in late M to early G1 phase. (TIF) [file pone.0151803.s002.TIF]

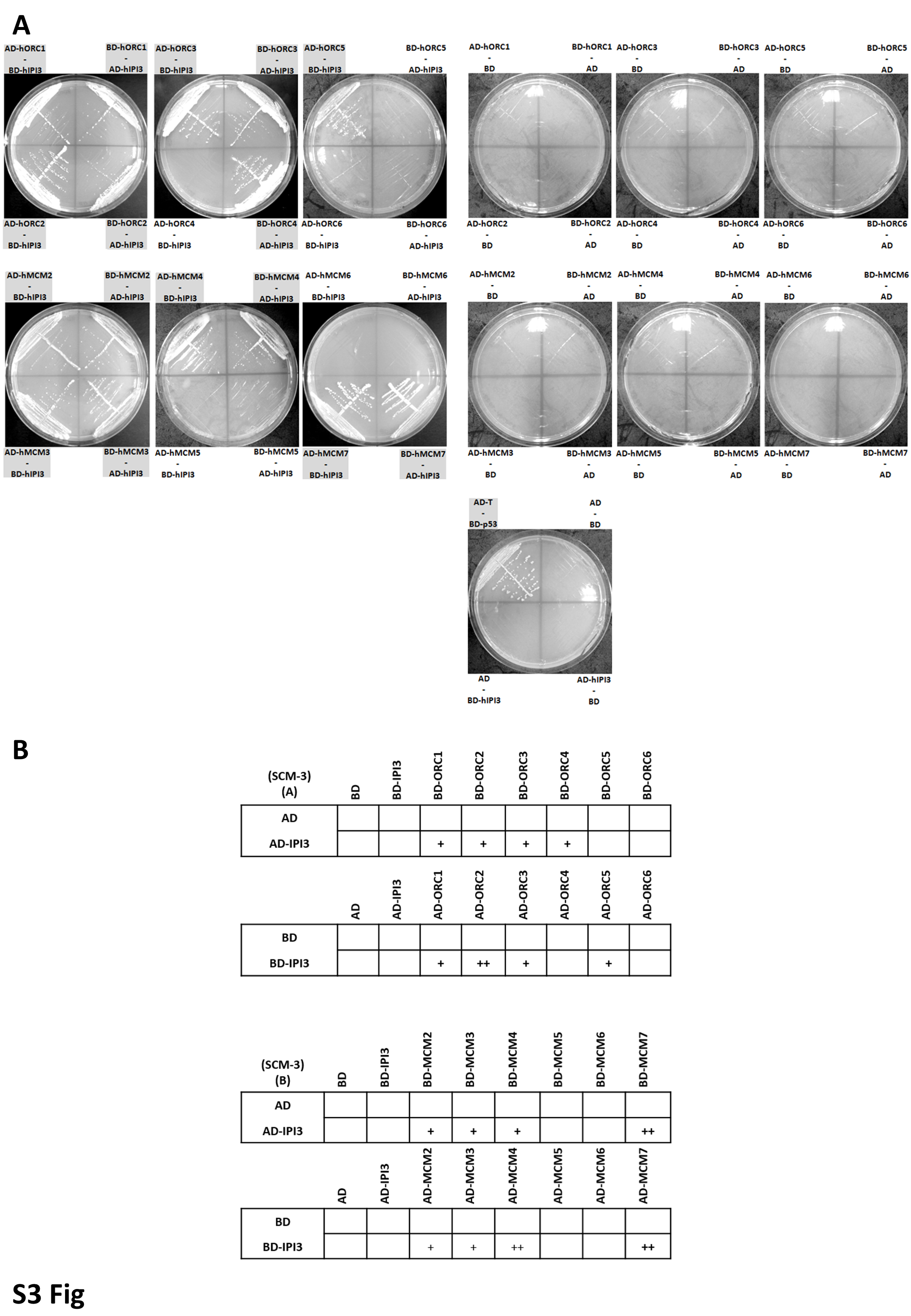

Supplement: S3 Fig — (A) AH109 cells transformants containing the indicated combinations of plasmids were streaked onto SCM-3 plates to examine the relative strength of the interactions. The combinations with positive interactions are marked by shades. (B) Summary of the results from (A). A single ‘+’ sign indicates a weak interaction while ‘++’ represents an intermediate interaction, relative to the strong interaction of the positive control which would be “+++”. Absence of the sign means no interaction. (TIF) [file pone.0151803.s003.TIF]

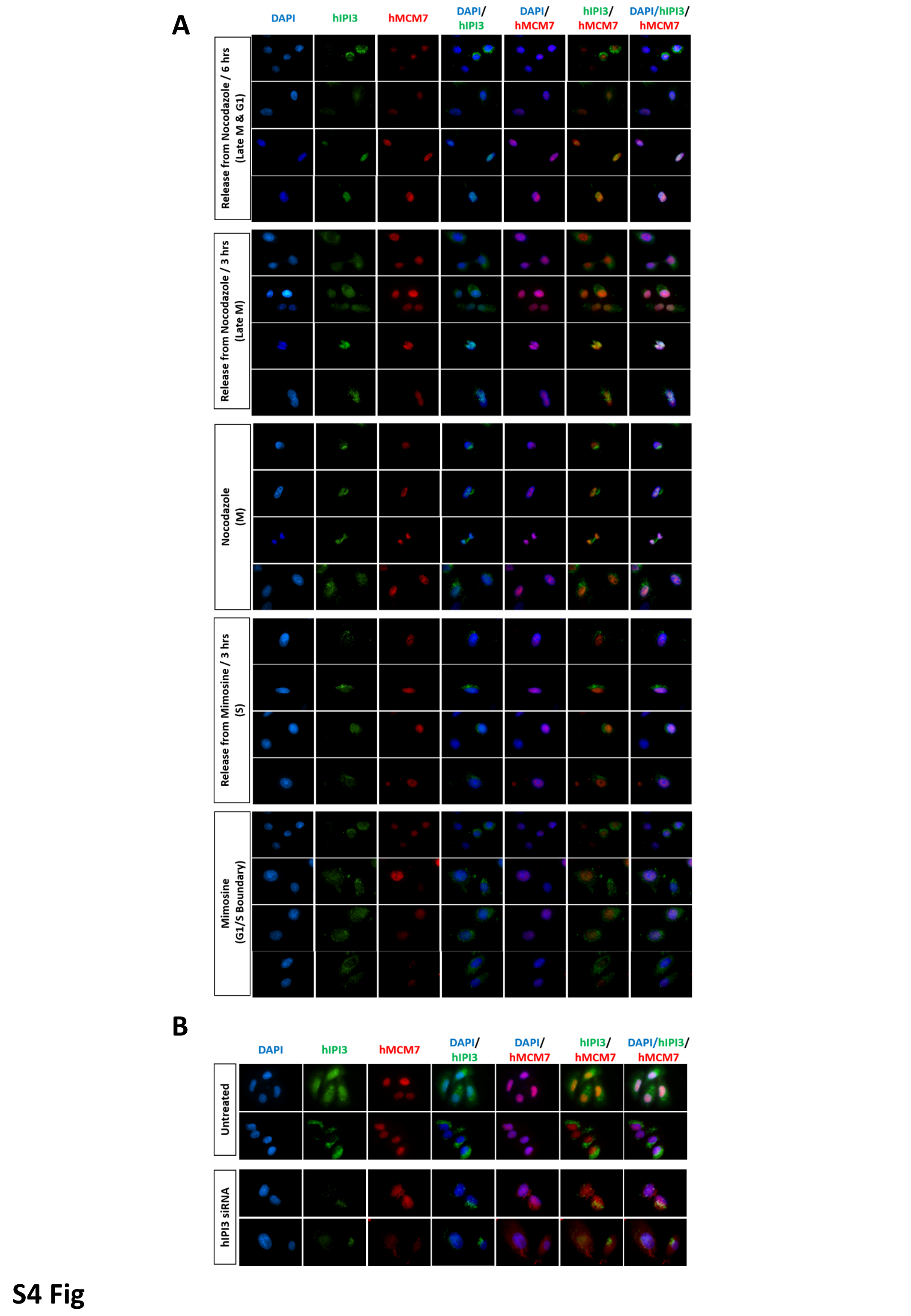

Supplement: S4 Fig — Photos in S4A Fig show more cells from the same experiment as shown in Fig 7A, and Photos in S4B Fig show more cells from the same experiment as shown in Fig 7B. (TIF) [file pone.0151803.s004.TIF]
